# Four Decades of Andean Timberline Migration and Implications for Biodiversity Loss with Climate Change

**DOI:** 10.1371/journal.pone.0074496

**Published:** 2013-09-11

**Authors:** David A. Lutz, Rebecca L. Powell, Miles R. Silman

**Affiliations:** 1 Center for Energy, Environment, and Sustainability and Department of Biology, Wake Forest University, Winston-Salem, North Carolina, United States of America; 2 Environmental Studies Program, Dartmouth College, Hanover, New Hampshire, United States of America; University of Sydney, Australia

## Abstract

Rapid 21st-century climate change may lead to large population decreases and extinction in tropical montane cloud forest species in the Andes. While prior research has focused on species migrations per se, ecotones may respond to different environmental factors than species. Even if species can migrate in response to climate change, if ecotones do not they can function as hard barriers to species migrations, making ecotone migrations central to understanding species persistence under scenarios of climate change. We examined a 42-year span of aerial photographs and high resolution satellite imagery to calculate migration rates of timberline–the grassland-forest ecotone–inside and outside of protected areas in the high Peruvian Andes. We found that timberline in protected areas was more likely to migrate upward in elevation than in areas with frequent cattle grazing and fire. However, rates in both protected (0.24 m yr^−1^) and unprotected (0.05 m yr^−1^) areas are only 0.5–2.3% of the rates needed to stay in equilibrium with projected climate by 2100. These ecotone migration rates are 12.5 to 110 times slower than the observed species migration rates within the same forest, suggesting a barrier to migration for mid- and high-elevation species. We anticipate that the ecotone will be a hard barrier to migration under future climate change, leading to drastic population and biodiversity losses in the region unless intensive management steps are taken.

## Introduction

The eastern slope of the tropical Andes contains high levels of species diversity and endemism [1], and is therefore a global conservation priority [2]. The steep elevation gradients in the Andes result in most species occupying relatively narrow elevational ranges, making forest species particularly sensitive to climate change [3]. In particular, shifts in temperature will require upslope migration for most species to remain in equilibrium with climate and therefore potentially avoid extinction [4], [5], [6]. This necessary migration becomes particularly acute given both the predicted temperature increases of 4–6°C by 2100 [7], [8], and that tree species in the region currently are showing a migration debt as a result of ongoing, contemporary climate change of >0.3°C per decade over the last half century [5], [7].

What is under-appreciated in most migration studies is the concept that ecotones – abrupt changes in species or plant functional types due to factors such as cloud base [9], soils [10], [11], or disturbance [12] – may not respond to climate change in the same way as individual species. Differential responses to climate change between species and ecotones may therefore result in barriers to migration, slowing the rate of species movement, or preventing migration altogether. In the tropical Andes, the major ecotone of interest in this context is the boundary between continuous closed-canopy forest and puna grassland, which here we refer to as “timberline,” following Young [13]. While this boundary only accounts for a small fraction of species range limits [14], species distribution modeling studies that incorporate climate change scenarios have shown that timberline stasis or low rates of timberline migration represent a major threat to Andean species with range centers above 2,000 meters [15]. Population sizes of cloud forest tree species are predicted to decrease in these models by a median of 70–85%, with many species eliminated completely, even when species themselves are capable of migration in equilibrium with climate [16]. Thus, the speed at which timberline migrates may be the single most important factor in determining the future population sizes for many Andean cloud forest species. Conservation strategies which attempt to promote upward migration of timberline [17] may therefore be important for climate change mitigation in the region.

The ecotone between closed canopy forest and puna grassland is set by both climatic and anthropogenic effects and has varied through time [12], [18], [19], [20], [21]. In the paleoecological record from the region, increased drying and the establishment of a natural fire regime after 12 kybp led to the expansion of puna grassland into the mid-Holocene dry period. Anthropogenic effects first appeared during the mid-Holocene dry period ca. 6 kybp which led to an increase in fire frequency and further depression in the timberline, thereby preventing an increase in the elevation of timberline in the late Holocene as wetter conditions returned to the puna [20]. Historically, intentional burning leading to the promotion of puna grassland from fires set for grazing [21], [22], [23], hunting [24], and intensified agriculture [25], [26], have lowered timberline by ∼500 m from where it would be in the absence of these anthropogenic disturbances [12], [19].

While the behavior of the grassland-forest ecotone is central to understanding the fate of species in the Andes under scenarios of climate change, both the basic rate of timberline movement and responses to temperature change and land management are unknown. We took advantage of two sets of high-resolution remotely sensed imagery separated by >40 years in the Southeastern Peruvian Andes and documented timberline migration in areas of differing levels of protection from disturbance. In particular, we asked: (1) what are the characteristics of timberline movement in terms of average overall rate and the proportion of timberline migrating? (2) Does protected area status affect Andean timberline movement? (3) What is the effect of starting elevation, a proxy for forest productivity, on movement of the grassland-forest ecotone?, and (4) How do rates of timberline advance compare to tree species migration rates measured in forests below the ecotone in the same valley? The study area forms the upper portion of an Andes-to-Amazon transect long-term ecological research site, providing a detailed understanding of biotic and abiotic effects on species ranges and ecosystem function [27], [28], paleoecological responses to climate change [29], and current species responses to climate change [5]. This context allows an integrated view of the effects of timberline movement on future Andean cloud forest biodiversity and ecosystem function.

## Methods

### Study Area

**Figure 1:**
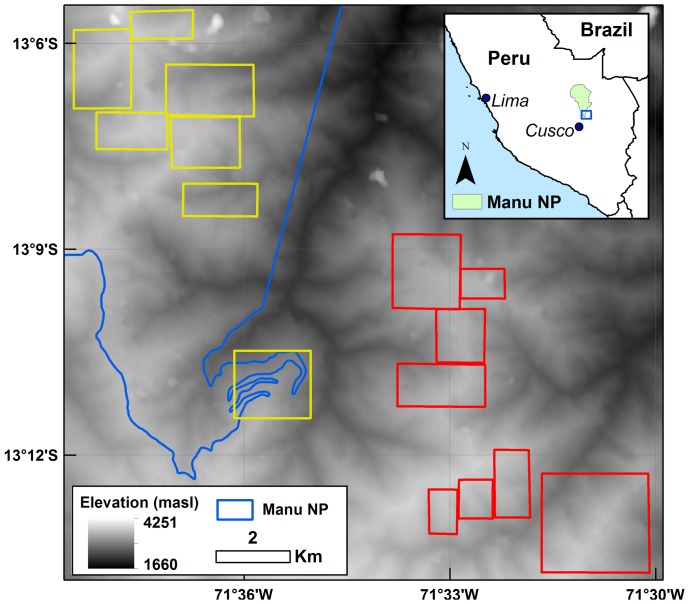
The study area. Areas highlighted represent the subsets of high resolution imagery analyzed; subsets in red indicate land under traditional management (unprotected), and subsets in yellow designate land that is managed for conservation (protected).

Our study area consisted of approximately 50 km2 in the southern area of Manu National Park (MNP) (11°51′23′’S, 71°43′17′’W) and the surrounding Kosñipata valley, on the east Andean slope at the headwaters of the Madre de Dios River in southeast Peru (Fig. 1). No specific permissions were needed for this study as all data collections were performed using remotely sensed imagery. There were no protected or endangered species involved with the image collection process. The ecotone between cloud forest and puna grassland is abrupt [13], [18], transitioning from forest (trees in the families Cunoniaceae, Melastomataceae, Rosaceae, and Rubiaceae with occasional stands of Chusquea bamboo [30]) to puna grassland that is dominated by tall tussock grasses in the genera Calamagrostis, Festuca, and Paspalum [30]. Shrubs in the families Asteraceae, Aquifoliaceae, Ericaceae, Escalloniaceae, and Melastomataceae, particulary the genera Escallonia and Brachyotum, are frequent in the less abrupt transition areas. This assemblage of species is similar to other grassland-forest ecotones within the Andes [31], and its boundary is easily identifiable from aerial imagery.

The study area was divided into two broad classes–protected and unprotected – based on land management regimes. Protected areas included land within the boundaries of MNP as well as land immediately adjacent to the Wayqecha Biological Station, a privately held conservation concession. Although under active conservation management, protected areas are still sometimes subject to disturbance, though at a lower rate than surrounding areas. Since 1974, the influence of grazing has been restricted in MNP; however, small numbers of cattle are permitted to seasonally graze within the protected area. Fire has been actively suppressed in the park, partially the result of monitoring efforts by park rangers and also due to the presence of natural fire breaks. Fires started outside the park still occasionally spread into the protected areas. The Wayqecha Biological Station on the southeastern border of MNP has been owned and managed by the Associación para la Conservación de la Cuenca Amazónica (ACCA) since 2003; grazing and fire disturbance have been minimized in the area. Timberline sampled within these protected areas ranged in elevation from 2,500 m to 3,700 m asl.

Unprotected areas included all privately owned land outside of the MNP and Wayqecha boundaries. These areas are currently managed by local communities and land is burned at intervals of 3 to 6 years to improve pasturage. Tussock grass species in the Andes respond favorably to fire [23] and once established can inhibit the establishment of cloud forest seedlings [22]. Timberline sampled within these unprotected areas ranged in elevation from 2,800 m to 3,700 m asl.

### Data

We compared the elevation of timberline between 1963 and 2005 by visual interpretation of historical panchromatic aerial photographs and high-resolution Quickbird satellite imagery. The imagery for this bitemporal study include: (a) Quickbird panchromatic imagery (0.6 m spatial resolution at nadir), acquired on May 5, 2005 and orthorectified by DigitalGlobe (Longmont, Colorado) and (b) aerial photographs collected by the United States Air Force’s Aerial Survey Teams during June, 1963, and digitized to 0.7 microns by the Peruvian Instituto Geographico Nacional. We clipped the aerial photographs and centered them on the timberline boundary in an effort to minimize the registration error introduced by extremely steep topography in the study area. Each subset was independently georegistered to the Quickbird image using 25 ground control points (rock outcrops, roads, isolated trees) and a first-order polynomial transformation with nearest neighbor resampling. Maximum root mean square registration error (RMSE) was 37 pixels (∼22 m), and the median RMSE for all subsets was 19 pixels (∼11.5 m). Subsets with RMSE greater than 50 pixels were excluded.

Elevations were determined using the Advanced Spaceborne Thermal Emission and Reflection Radiometer (ASTER) Global Digital Elevation Model (GDEM) version 2 (spatial resolution of 1 arc-second), developed jointly by the Japanese Ministry of Economy, Trade, and Industry (METI) and the U.S. National Aeronautics and Space Administration (NASA). We projected the ASTER GDEM 2 to Universal Transverse Mercator (UTM, Zone 19 S) to correspond to the imagery datasets, resampling the GDEM to 30-m spatial resolution. Overall absolute vertical accuracy of the GDEM2 product is reported to be 8.86 m; horizontal errors are subsumed within this vertical error assessment [32]. The GDEM2 elevation includes the height of aboveground features (i.e., tree canopy) in addition to ground elevation; therefore, relative differences of elevation between grid cells should be more accurate than absolute elevation estimates.

### Analysis

**Figure 2:**
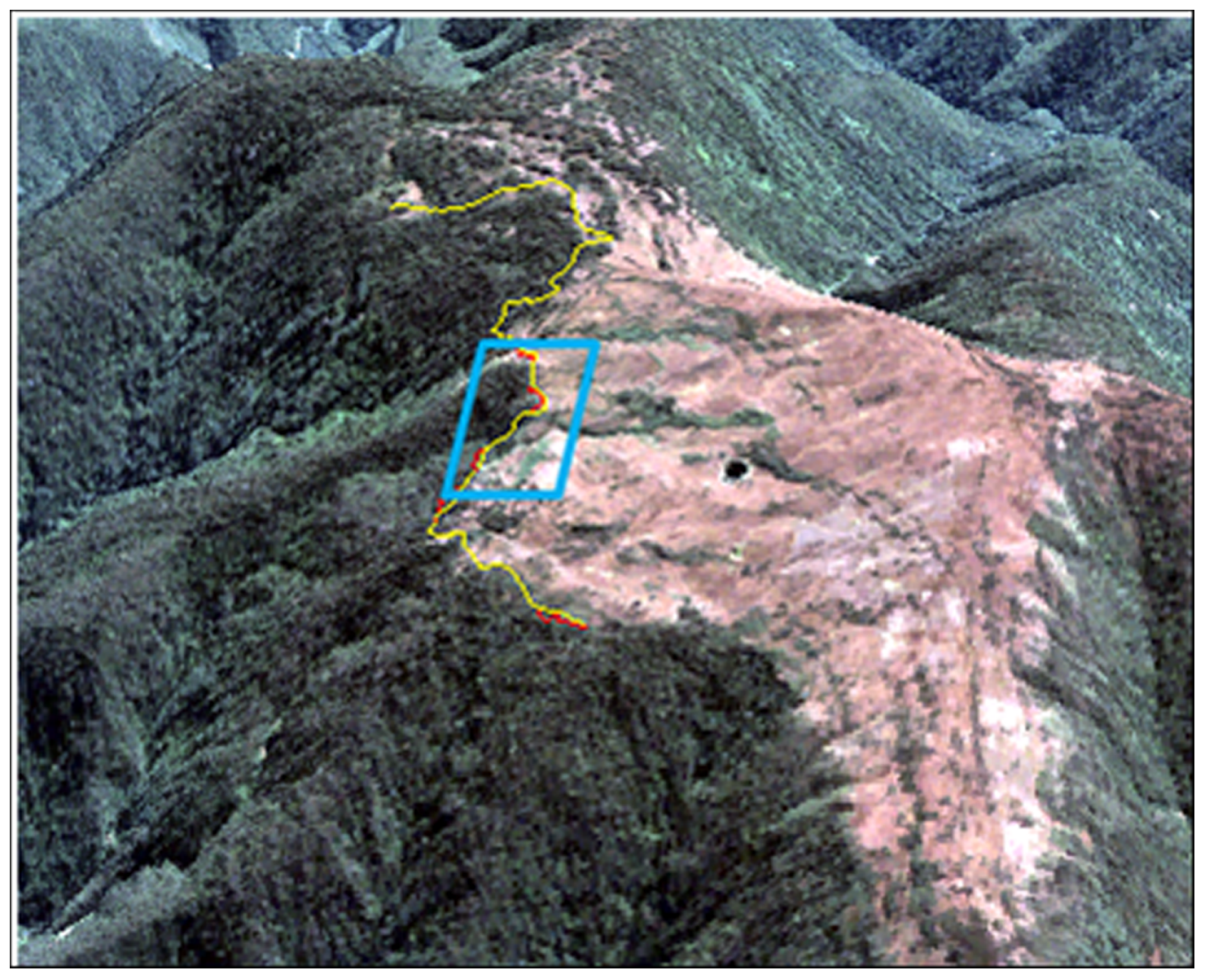
Identification of timberline using remotely sensed imagery. Digitized 1963 timberline (yellow line) draped over the ASTER GDEM2. A 2005 Quickbird multispectral true color composite serves as the background layer. The blue polygon highlights the timberline subset displayed in Fig. 3; red segments indicate change in timberline elevation between 1963 and 2005.

**Figure 3:**
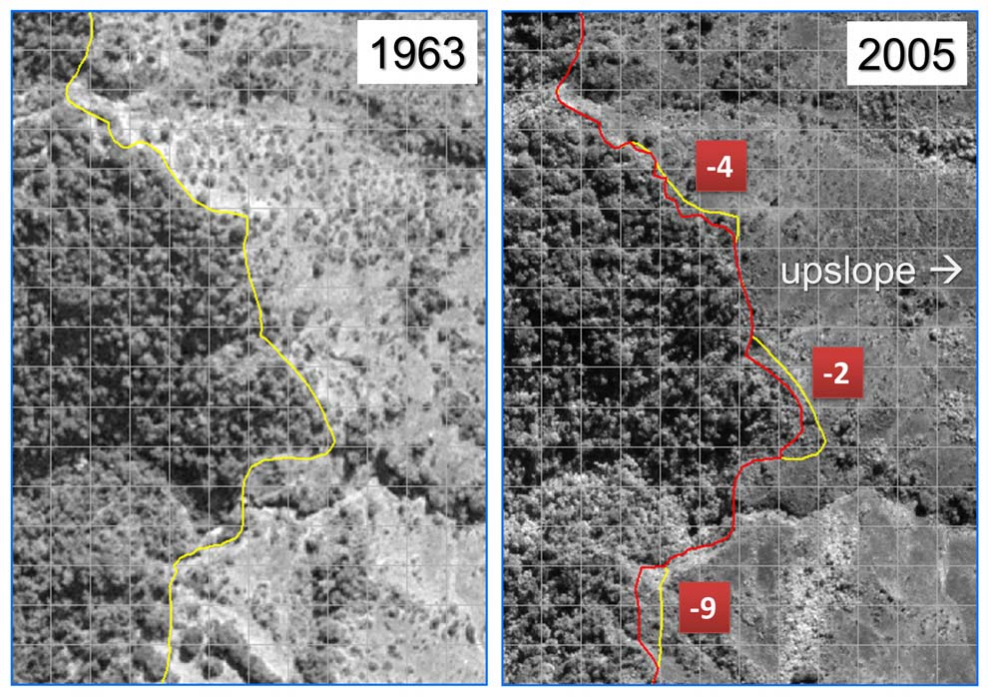
Calculation of timberline movement. An example of a 1963 aerial photograph (left) and 2005 Quickbird panchromatic imagery (right) of the same location in the study area. The yellow line delimits 1963 timberline and the red line delimits 2005 timberline. Differences between mean segment elevations (m) are indicated in red squares. Grid cells correspond to the 30-m GDEM cell boundaries.

The spatial resolution of the imagery allowed for the identification of individual tree crowns, offering the ability to discriminate timberline with high confidence. We delineated timberline, defined as the boundary of continuous tree crown cover [13], [33], rather than the maximum elevation of individual trees. The timberline boundaries for each date were manually digitized and converted to a shapefile for analysis in ArcGIS software [34]. To ensure that this manual classification method was robust, we compared our delineation to that of a linear support vector machine (SVM) technique and found similar results [35]. The elevation of each 30-m DEM cell intercepted by timberline was extracted and recorded, generating empirical distributions of timberline elevation for each date and under each management regime. To ensure that no topographic artifacts or anomalies from the DEM were included in the analysis, we draped each image subset and its corresponding shapefile over a 3D rendering of the DEM using the 3D SurfaceView function in ENVI software [36](Fig. 2). Each digitized timberline was partitioned into change and no-change segments. For those segments that migrated between 1963 and 2005, mean segment elevation for each date was calculated as the mean value of the DEM cells intercepted by the segment. Mean elevation change for each segment was calculated as the difference between the 2005 segment mean and the 1963 segment mean (Fig. 3). Distributions of timberline elevation change were generated by assigning the segment mean elevation change value to every 30-m DEM cell intercepted by the segment, both inside and outside protected areas. Cells intercepted by both the 1963 timberline and the 2005 timberline were assigned a change value of zero.

Timberline dynamics between 1963 and 2005 were analyzed in terms of the percent change of the total length measured and the direction and magnitude of that change. We calculated mean rates of timberline movement for the entire length of timberline delineated, as well as for those segments that had migrated. All estimates were calculated independently based on protection status. Rates of change were compared to current rates of observed tree migration on the eastern slope of the southeastern Peruvian Andes [5], as well as estimated rates of migration since the Holocene [29]. Using these empirical estimates of timberline migration rates, we calculated the number of years that would be needed for timberline to reach equilibrium with climate projections for the year 2100. Finally, for those timberline segments that had migrated between the two dates, we investigated the relationship between starting elevation, protection status, and the magnitude of elevation change using the following generalized linear model (GLM):
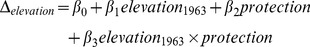
(1)where Δ elevation was the change in mean segment elevation between 1963 and 2005, elevation1963 was the mean starting elevation of the segment, protection represented the protected status of the segment in 2005, elevation1963 × protection represented the interaction between the status and starting elevation effects, and βi represented the estimated parameters.

We also looked at migration rates and the effects of protection status nonparametrically using bootstrapped samples of means and differences between treatments by taking 20,000 bootstrap samples of the means of timberline elevation in protected areas, unprotected areas, and the difference between protected and unprotected areas in R statistical software [37]. The resulting distributions were a nonparametric distribution of the mean annualized rates of protected and unprotected status and the difference between the rates (treatment effect).

## Results

### Changes in Timberline, 1963–2005

Between 1963 and 2005, nearly 80% of the timberline delineated in the study area remained stable; often, the same individual tree crowns could clearly be identified in both dates (Fig. 3). In areas that are currently protected, we delineated 40.8 km of timberline in the 1963 imagery; by 2005, 29% of that length (∼11.7 km) had migrated in a direction of increasing elevation, and less than 1% (∼0.3 km) had moved to lower elevations (Table 1). In unprotected areas, we delineated 41.5 km of timberline in the 1963 imagery; by 2005, approximately 8.6% of this timberline length (∼3.6 km) had migrated to higher elevation, while 4.5% (∼1.9 km) had moved to lower elevations (Table 1).

**Table 1:** Summary of timberline change between 1963 and 2005.

Status	Mean 1963elevation	Total 1963 length(km)	Up change(km)	Down change(km)	Total % change	Up %change	Down % change	Mean elevation change (m)
Protected	3,267	40.8	11.7	0.3	29.5	28.7	0.8	+10
Unprotected	3,327	41.5	3.6	1.9	13.1	8.6	4.5	+2

**Figure 4:**
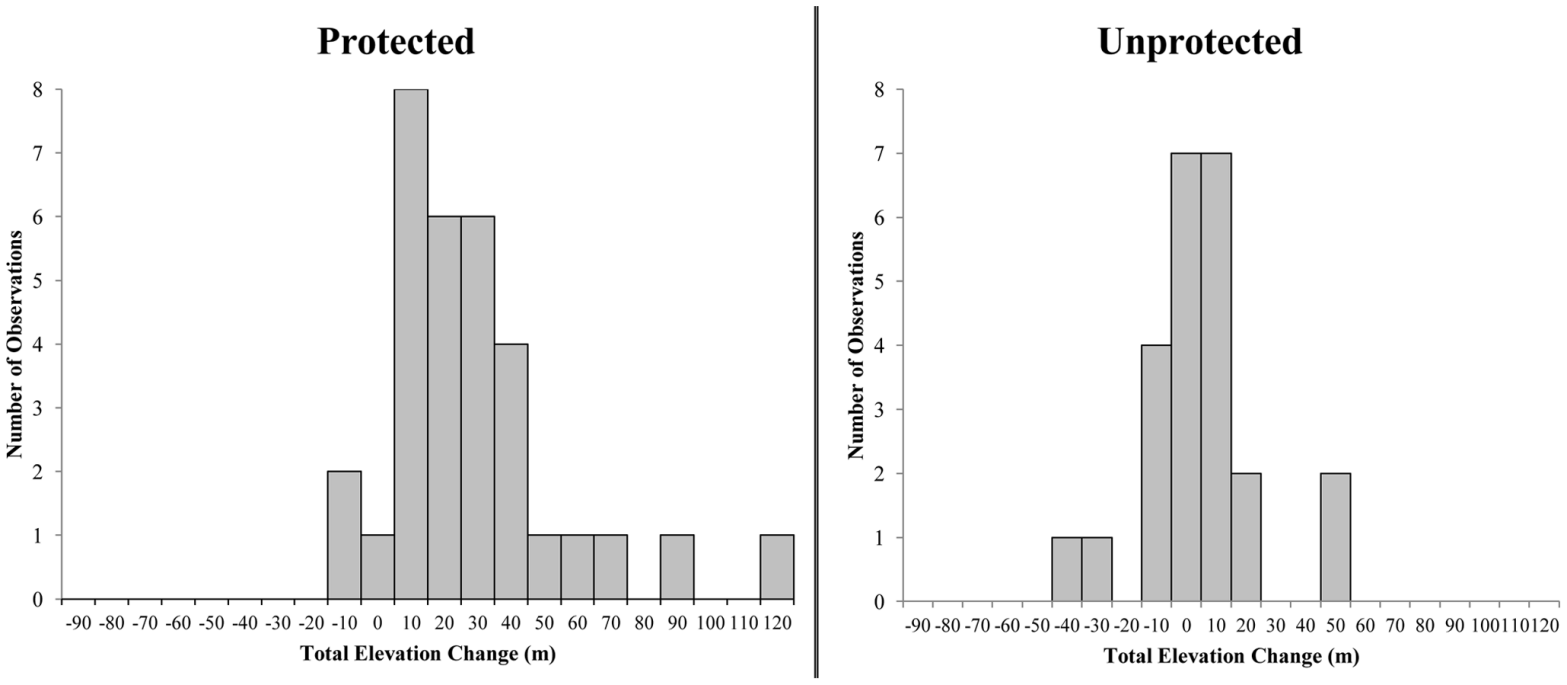
Migration of timberline in protected and unprotected areas. A graph showing the distribution of mean elevation change for timberline segments that migrated between 1963 and 2005 in protected areas (*n = *32) and unprotected areas (*n = *24). Mean elevation change for segments inside protected areas was significantly greater than zero (*one-sided t-test, t* = 5.01, *df* = 31, *p*<0.0001). Outside protected areas, a given change segment was equally likely to migrate up as to migrate down in elevation; the mean of elevation change for all segments in unprotected areas was not significantly different than zero (*two-sided t-test, t* = −0.13, *df* = 23, *p* = 0.90). Migration was more likely to occur in an upslope direction in protected areas compared to non-protected areas (*Fisher’s Exact Test*, *df* = 1, *p*<0.0003).

Within protected areas, a total of 29 timberline segments migrated upwards and only 3 segments migrated downwards. In unprotected areas, 11 segments migrated upwards and 13 migrated downwards (Table 2, Fig. 4). For a given segment of timberline, regardless of segment length or starting elevation, the probability of upward migration was substantially greater in protected areas. Mean elevation change for segments inside protected areas was significantly greater than zero (one-sided t-test, t = 5.01, df = 31, p<0.0001). Outside protected areas, a given change segment was equally likely to migrate up as to migrate down in elevation; the mean of elevation change for all segments in unprotected areas was not significantly different than zero (two-sided t-test, t = −0.13, df = 23, p = 0.90). Migration was more likely to occur in an upslope direction in protected areas compared to non-protected areas (Fisher’s Exact Test, df = 1, p<0.0003).

**Table 2:** Summary of timberline segments that migrated between 1963 and 2005.

Status	Mean 1963elevation	Up segments(count)	Down segments(count)	Mean ± SD segmentlength (m)	Unweighted meanelevation change (m)*	Weighted mean elevation change (m)**
Protected	3,267	29	3	376±273	+23.5	+33.1
Unprotected	3,327	11	13	226±291	−0.50	+13.8

* Mean elevation of segments weighted equally.

** Mean elevation of segments weighted by 1963 segment lengths.

**Figure 5:**
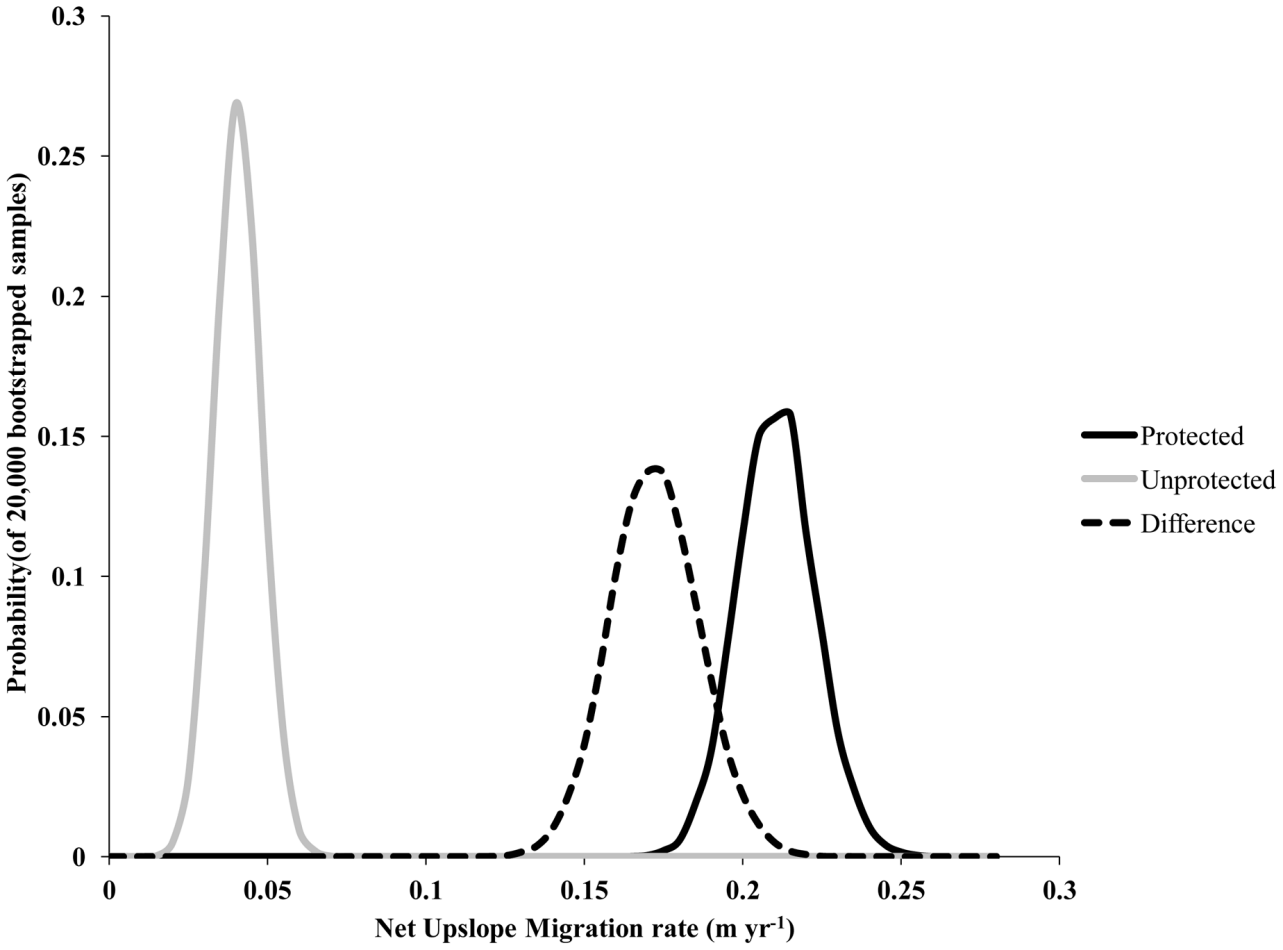
Bootstrapping of timberline migration rates. Bootstrapped sample probability of mean yearly migration rate for the entire length of measured timberline in protected and unprotected areas (∼40 km in each area), and differences between these treatments. Total samples equaled 20,000.

Migration of timberline was extremely patchy on the landscape, regardless of protection status. The mean 1963 length (±1 SD) of segments that migrated, regardless of direction, was 376±273 m in protected areas and 226±291 m in unprotected areas (Table 2). The mean overall movement of timberline was+0.24 m yr^−1^ (+10 m total) in protected areas and +0.05 m yr^−1^ (+2 m total) in unprotected areas (Table 3). If we consider only those segments of timberline that migrated during the study period, average elevation change was +0.79 m yr^−1^ (+33 m total, 30% of observed timberline length) in protected areas and +0.33 m yr^−1^ (+14 m total, 13% of observed timberline length) in unprotected areas (weighted relative to 1963 timberline length; Table 3). Bootstrap analysis of the entire timberline showed that the mean annual migration rate of 0.208 m yr^−1^ (s.e. = 0.00009) for protected areas, 0.038 m yr^−1^ (s.e. = 0.00005) for unprotected areas, with a difference in annualized rate of 0.170 m yr^−1^ (s.e. = 0.0001) (Fig. 5).

**Table 3:** Annualized migration rates (in *vertical* m yr^−1^) and number of years required to reach equilibrium with 2100 climate projections.

Status	Annualized migration rates (m y^−1^)		Years to 2100 climate equilibrium	
	Change segments	Timberline	Change segments	Timberline
Protected	0.79	0.24	1,140	3,750
Unprotected	0.33	0.05	2,730	18,000

### Timberline Equilibrium with Climate Change

We calculated the number of years that would be needed for timberline to reach equilibrium with projected climate in 2100. We followed [16], who assumed a 5°C warming over the next century, estimates which align with projections regional climate models [8]. Assuming no additional changes in climatic factors other than temperature, timberline would need to migrate ∼900 m in elevation. Given the mean rates of timberline movement that we documented over the 42-year interval, a migration of 900 m in altitude would take 3,750 years in protected areas and 18,000 years in unprotected areas to keep pace with climate change of this magnitude (Table 3). If we apply the annualized rates for only the areas of timberline observed to be migrating, we would expect timberline to take 1,140 yrs to migrate 900 m in elevation in protected areas and 2,730 yrs in unprotected areas. However, this would be only 30% and 13% of the total timberline length, respectively, with the remainder being static.

### Combined Effects of Protected Status and Elevation on Timberline Migration

**Figure 6:**
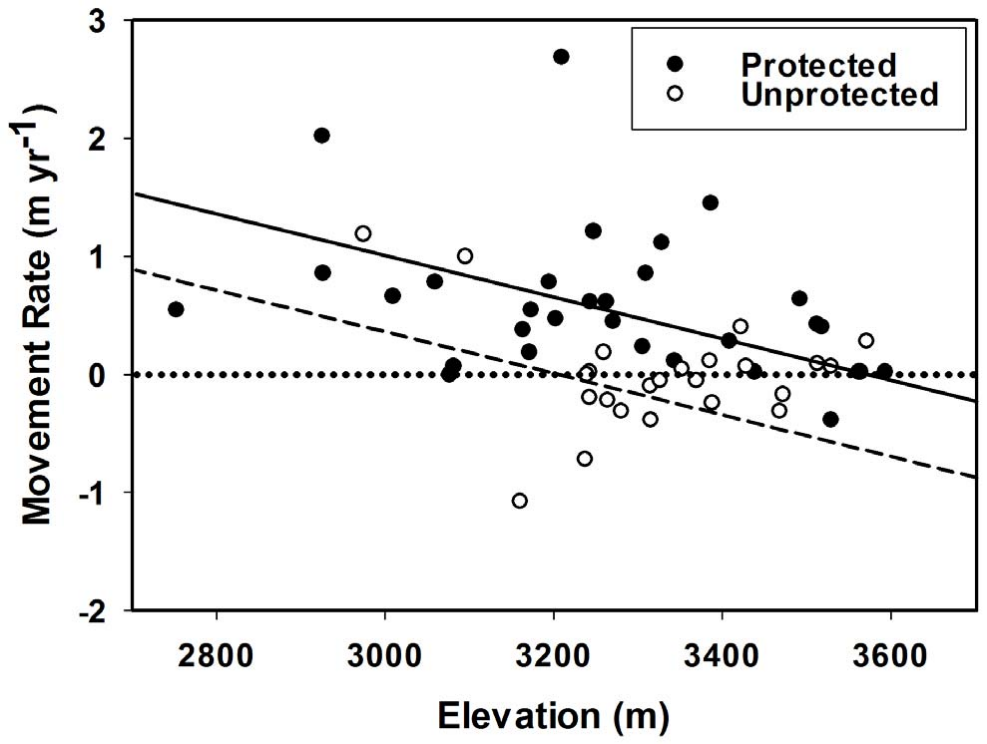
Influence of elevation upon upslope timberline migration rate. A graph showing the rate of mean elevation change versus starting elevation for segments that migrated during the study period. Filled circles represent samples in protected areas; unfilled circles represent those samples in unprotected areas. Mean elevation was predicted as a function of segment starting (1963) elevation, protection status, and the interaction between the two effects.

For migrating timberline segments, the initial 1963 elevation influenced the net change in elevation (Fig. 6; GLM full model, adjusted-R2 = 0.342, F = 10.65, df = 3,52, p<0.0001). The rate of timberline migration was higher in protected areas than non-protected areas (GLM protection, F = 13.1, p = 0.01) and decreased with increasing elevation (GLM elevation1963, F = 6.98, p<0.001). The effect of elevation on timberline migration rate was independent of protected area status (GLM elevation1963 × protection interaction, F = 1.24, p = 0.27). There was no significant difference between mean starting elevation for migrating timberline segments based on protection status (two-sided t-test, t = 1.21, df = 54, p = 0.23); mean 1963 elevation (±SE) was 3,267±32 m for protected areas and 3,327±37 m for unprotected areas.

## Discussion

The grassland-forest ecotone showed remarkable stability over the 42 yr study period, with timberline remaining stable in nearly 80% of the landscape. While other studies of high elevation timberline have shown marked migration at rates up to 1.8 m yr^−1^ [38], the ecotone in our study migrated at an overall rate of between 0.05 and 0.24 m yr^−1^, with all the migration concentrated along just ∼20% of its length. This observed migration is only 0.9–4.3% of that required to remain in equilibrium with ongoing observed temperature change in the region, and only 0.5–2.3% of that required to remain in equilibrium with predicted climate change in the region through 2100 assuming the rate used by [16]. To put is another way, at current rates of migration in unprotected areas, it would take timberline 18,000 yrs to reach an elevation where the ecotone would be in equilibrium with temperatures predicted for 2100.

While timberline migration in the region is slow, the migration of forest trees is not. Feeley et al. found that trees in forests below timberline in the same valley were migrating at rates between 0–22 m yr^−1^, with average migration rates ranging from 3–7 m yr^−1^ [5]. Based on that study, the average rate of movement for forest taxa is 12.5 times faster than the ecotone, with the fastest moving taxa outpacing ecotone movement by 110 times. This difference between species and ecotone migration rates will press species against the grassland-forest boundary, forming a barrier to forest species migration. The effect will be potentially large decreases in Andean cloud forest tree species population sizes and likely extinctions for our study area under current rates of movement [15]. The measurement of timberline movement and forest species migration in the same study area in response to the same climatic forcing provides a complementary view of the challenges involved in managing high biodiversity Andean landscapes under scenarios of climate change.

### Factors Controlling the Pace of Timberline Migration

The pace of timberline movement in areas where timberline migrated was not constant across the landscape, showing differences due to protected area status of the land and the elevation of timberline at the start of the study interval. Protected area status increased the migration rate of the grassland-forest ecotone by ∼5 times in these areas, taking it from being nearly static (migrating at 0.05 m yr^−1^, on average, with only 13% of the length of the timberline moving) to migrating at 0.24 m yr^−1^ with 30% of timberline migrating. When we tested the influence of protected status nonparametrically, we came to the same conclusion. Considering that Manu National Park was established 11 years into the study interval, migration rates were likely the same as in unprotected areas, ∼0.3 m yr^−1^, during the period 1963–1974, with the 30% of timberline that was mobile shifting at a rate of ∼1.0 m yr^−1^ during the protected interval (1974–005). While this would still be less than half the rate of observed forest species migration in the same area, and only ∼10% of that necessary to keep pace with predicted temperature change by 2100, it would be similar to the rates observed in the paleorecord from the region [29], which range between 0.2 and 0.7 m yr^−1^ during the Pleistocene-Holocene transition. The correspondence between our data and Quaternary migration rates indicate that timberline was able to remain in equilibrium with climate as it had in the past and that slow rates observed when fire and grazing are controlled in the landscape do not reflect an inherent disconnect in this system between species and ecotone migration rates. Rather, they reflect the unprecedented pace of predicted temperature change as compared to the late Quaternary, and the resulting decoupling of ecosystem responses to climate change. The transient phase in which high elevation ecosystems adjust to climate change is likely to reflect states with no analog in the paleorecord, at least in the late Quaternary.

Elevation of the grassland-forest ecotone was a significant factor controlling the migration rate of timberline over the study period. Migration rates were highest for migrating segments at the lowest elevations and decreased with increasing starting elevation, changing 5-fold over the ∼1 km elevation difference encompassed by the study. Migration rates are likely highest at lower elevations for several reasons. First, forest productivity decreases strongly with increasing elevation. This means that forest regrowth will have a relative advantage at lower elevations. Second, when selective browsing occurs, grass can out-compete seedlings that are grazed upon thus limiting an increase in tree cover. This also occurs when repeated burning of grasses creates a “fire trap” that kills woody regeneration yet which does not inhibit re-growth by grasses. As woody productivity decreases with increasing elevation in this system, even long fire return intervals may suppress woody regeneration, maintaining dominance by grasses. Grazing can increase the spread of woody seedlings by decreasing the biomass of grasses, and thereby the ability of grassland to fuel fire. However, in this system, cattle both browse and graze, and the relative effect of cattle is unknown as compared to fire. In all cases, the lower tree productivity at higher elevation will be a disadvantage to timberline movement in the highest regions of the study area.

Another possible ecophysiological mechanism for limited timberline migration involves the abiotic factors influencing competition between grass and tree seedlings at high elevations. High solar radiation levels at high elevations have inhibited growth in most forest seedlings when they were placed in unshaded grassland [31]. Exposure to low overnight temperatures and night-time sky also poses additional limitations to tree seedling establishment at timberline [39], [40], particularly when coupled with high levels of sunlight. These factors place tree seedlings at another competitive disadvantage compared to the tussock grasses in puna. While more specific field experiments are necessary to fully understand the physiological constraints on seedling establishment at timberline in our study area, and in general [41], our results indicate that Andean cloud forest trees within closed canopy at lower elevations in this region of Peru are likely to migrate more swiftly than those at the timberline ecotone at high altitude.

### Can the Timberline Ecotone Migrate Faster?

While Manu National Park and the adjacent privately held reserve have protected status, they are by no means fully protected from disturbances such as fire and cattle grazing. The area inside of MNP was subject to the same types of disturbance in the period 1963–1974 as those outside the protected areas today. Large blackened areas on the landscape are visible inside the park in the 1963 photographs, which were taken at the peak of fire season. Cattle were grazed in the protected areas by both the Bravo family (Bravo, personal communication) and communities in the Mapacho river valley which abuts the southern border of the park. The latter group still seasonally grazes cattle in the high elevation of the park. Both of these factors point to the potential of timberline to migrate faster than the observed rates.

Warming itself is likely to accelerate the rate of movement of timberline. Because timberline generally migrated more quickly at lower elevations than at higher elevations, likely due to increased tree productivity and growth rates with warmer temperature, we expect both that warming temperatures will accelerate the rate of migration in the future, and that acceleration, or the potential for rapid migration, will increase as the timberline becomes farther out of equilibrium with temperature. Indeed, at the lowest elevations, timberline segments were observed to advance at 0.71 m yr^−1^, nearly five times the average rate of movement. An essential condition for any movement of timberline, however, is active fire management and a decrease in fire return time. If fires increase in frequency, a real possibility with increasing temperatures, and in the absence of fire management, timberline will remain static or decrease in elevation.

The competitive interactions between grass and tree species, either directly through competition for resources or indirectly through the effects of fire, are a major factor preventing the advance of the timberline ecotone. While we did not perform a quantitative analysis on the effect of landslides in our study area, we did fortuitously observe several instances in which landslides occurred across timberline. While all landslides visible in 1963 were fully recolonized and filled with tree crowns by 2005, we also looked at short term dynamics by comparing the 2005 Quickbird image with a 2009 Quickbird image of our study area. In two cases, a landslide occurring in 2005 had been recolonized by trees by 2009 in contrast to the slow recolonization of puna grassland areas by forest. Recent field collections within some of these and other nearby landslides, which exposed parent rock material, also indicate that trees and shrubs may colonize the areas within just two years (Clark, KL, personal communication). Thus, while landslides may initially depress timberline, they may simultaneously provide avenues for rapid upslope migration of tree species. This suggests that actively disturbing the soils along the grassland-forest ecotone might lead to similar rapid advancement of timberline, though only if fire is managed in the landscape.

## Conclusions

Long-term observations of tropical timberline are largely absent from the scientific literature and our study presents insight into the behavior of this ecotone with respect to future impacts of climate change and management regimes. Timberline in our study area remained mostly stable, despite climatic warming in the region. Using the borders of two protected conservation areas as a natural experiment, we find that limiting grazing pressure and fire result in increased upslope migration rates of forests, though these rates remain far below the velocity needed to for timberline to remain in equilibrium with projected climate warming. Furthermore, upslope timberline migration was significantly limited at higher elevations compared to lower elevations.

Our observations of the slow migration of the grassland-forest ecotone supports the most dire projections for species and Andean cloud forest population losses with climate change (−53% to ^−^96% according to [16]). The cloud forest that we studied constitutes part of the Andes Biodiversity Hotspot and if these losses are realized, a massive reduction of tropical montane biodiversity in the region would result. While conservation efforts have thus far sought to obtain land holdings and limit disturbance in the form of grazing and fire, our data suggest that these efforts are not sufficient by themselves. More intensive management to promote migration of cloud forest tree seedlings may be necessary to minimize population losses in the upcoming century. Observational evidence from our imagery and corroborating field data indicate that landslide disturbances may provide avenues for rapid timberline migration. Further field investigations on the behavior of tree seedlings in puna grassland will help elucidate both biotic and abiotic controls over migration, as well as provide guidance for adaptive management. This experimentation will be necessary to ensure that future management actions would result in acceptable conservation outcomes.
